# Supplementary material for: A 14 immune-related gene signature predicts clinical outcomes of kidney renal clear cell carcinoma
Source: PeerJ. 2020 Oct 29;8:e10183. doi: 10.7717/peerj.10183 (PMC7603789; doi:10.7717/peerj.10183)
Supplement: Supplemental Information 1 [file peerj-08-10183-s001.docx]

| **signatures** | **genes** | **frequency** |
| --- | --- | --- |
| 14_genes | ENSG00000205809;ENSG00000108688;ENSG00000075213;ENSG00000010319;ENSG00000184451;ENSG00000183682;ENSG00000158815;ENSG00000223802;ENSG00000113520;ENSG00000104826;ENSG00000084652;ENSG00000169083;ENSG00000174564;ENSG00000015475 | 282 |
| 15_genes | ENSG00000205809;ENSG00000108688;ENSG00000075213;ENSG00000010319;ENSG00000184451;ENSG00000183682;ENSG00000158815;ENSG00000223802;ENSG00000180875;ENSG00000113520;ENSG00000104826;ENSG00000084652;ENSG00000169083;ENSG00000174564;ENSG00000015475 | 216 |
| 13_genes | ENSG00000205809;ENSG00000108688;ENSG00000075213;ENSG00000010319;ENSG00000184451;ENSG00000213903;ENSG00000158815;ENSG00000223802;ENSG00000113520;ENSG00000104826;ENSG00000169083;ENSG00000174564;ENSG00000015475 | 192 |
| 16_genes | ENSG00000205809;ENSG00000108688;ENSG00000075213;ENSG00000010319;ENSG00000184451;ENSG00000183682;ENSG00000114646;ENSG00000158815;ENSG00000223802;ENSG00000180875;ENSG00000113520;ENSG00000104826;ENSG00000084652;ENSG00000169083;ENSG00000174564;ENSG00000015475 | 162 |
| 12_genes | ENSG00000205809;ENSG00000108688;ENSG00000075213;ENSG00000010319;ENSG00000184451;ENSG00000213903;ENSG00000158815;ENSG00000113520;ENSG00000104826;ENSG00000169083;ENSG00000174564;ENSG00000015475 | 135 |
| 17_genes | ENSG00000205809;ENSG00000108688;ENSG00000075213;ENSG00000010319;ENSG00000184451;ENSG00000173578;ENSG00000183682;ENSG00000114646;ENSG00000158815;ENSG00000223802;ENSG00000180875;ENSG00000113520;ENSG00000104826;ENSG00000084652;ENSG00000169083;ENSG00000174564;ENSG00000015475 | 9 |
| 18_genes | ENSG00000205809;ENSG00000204577;ENSG00000108688;ENSG00000075213;ENSG00000010319;ENSG00000184451;ENSG00000173578;ENSG00000183682;ENSG00000114646;ENSG00000158815;ENSG00000223802;ENSG00000180875;ENSG00000113520;ENSG00000104826;ENSG00000084652;ENSG00000169083;ENSG00000174564;ENSG00000015475 | 4 |

TableS1: summary of signatures and gene groups with frequency of elastic-net
