# Supplementary figures and images for: A 14 immune-related gene signature predicts clinical outcomes of kidney renal clear cell carcinoma
Source: PeerJ. 2020 Oct 29;8:e10183. doi: 10.7717/peerj.10183 (PMC7603789; doi:10.7717/peerj.10183)

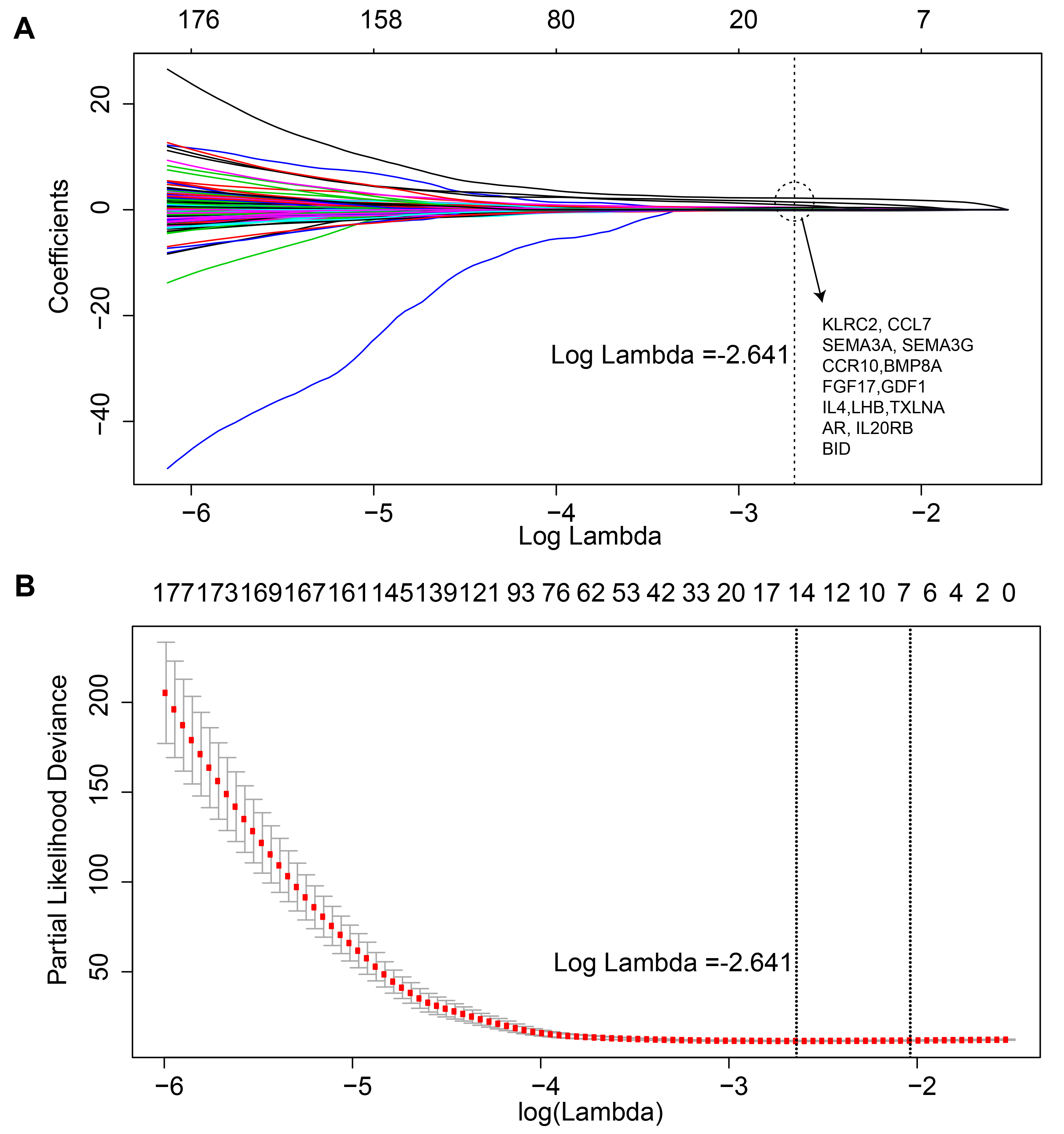

Supplement: Supplemental Information 2 — (A) LASSO coefficient values of the 14 immune related genes. (B) L1-penalty of cox regression. [file peerj-08-10183-s002.jpg]

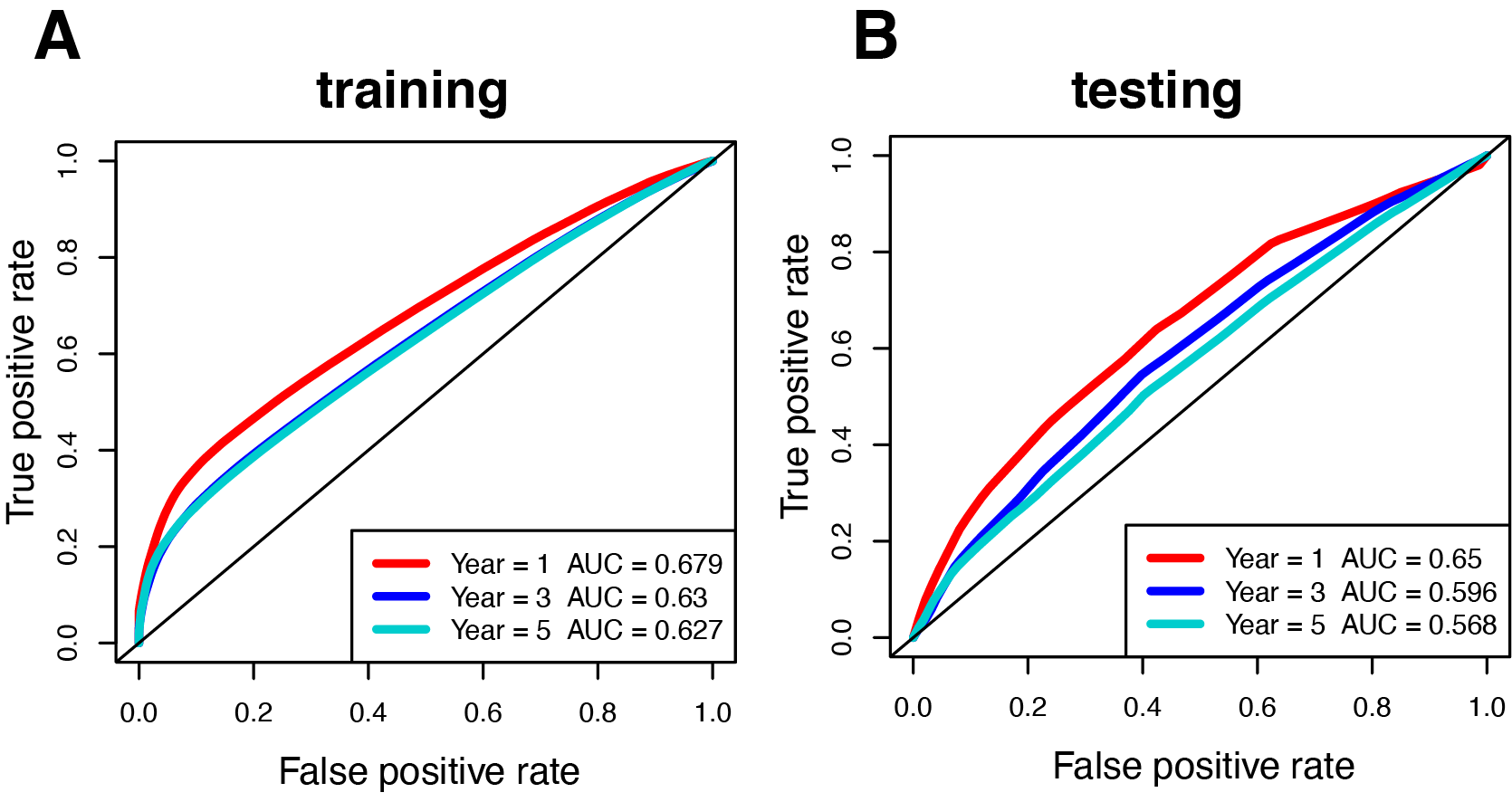

Supplement: Supplemental Information 3 — (A-B) The AUC values of 1, 3, and 5 year in the two data sets are 0.679, 0.63, 0.627 and 0.65, 0.596, 0.568, respectively. [file peerj-08-10183-s003.png]

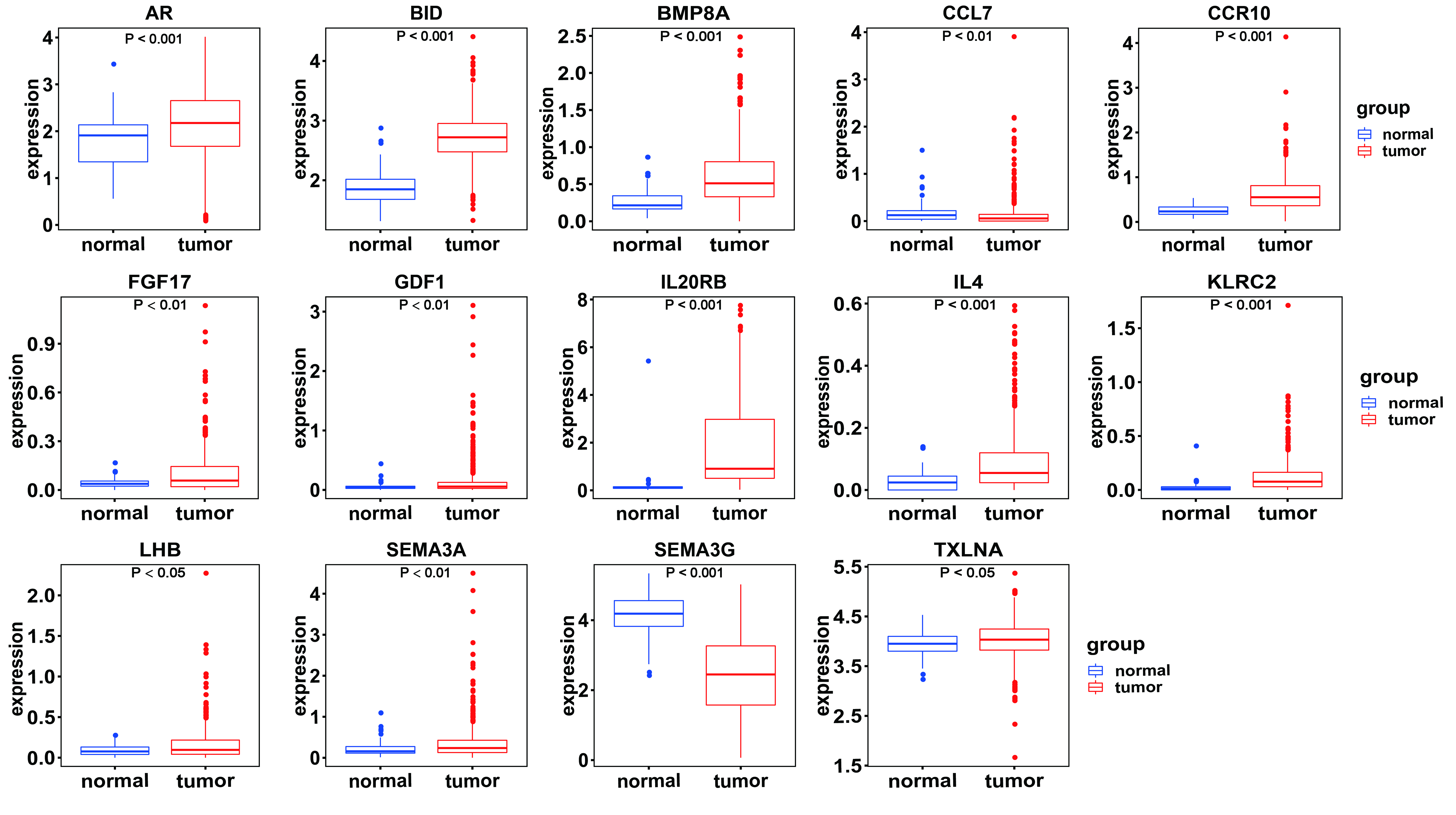

Supplement: Supplemental Information 4 [file peerj-08-10183-s004.jpg]
